# Unique high-temperature tolerance mechanisms of zoochlorellae *Symbiochlorum hainanensis* derived from scleractinian coral *Porites lutea*

**DOI:** 10.1128/mbio.02780-23

**Published:** 2024-02-22

**Authors:** Yilin Xiao, Luyao Gao, Zhiyong Li

**Affiliations:** 1 Marine Biotechnology Laboratory, State Key Laboratory of Microbial Metabolism, School of Life Sciences and Biotechnology, Shanghai Jiao Tong University, Shanghai, China; Corporación CorpoGen, Bogotá D.C., Colombia

**Keywords:** *Symbiochlorum hainanensis*, high-temperature tolerance, antioxidation, photosynthesis homeostasis, selenate reduction

## Abstract

Global warming is a key issue that causes coral bleaching mainly because of the thermosensitivity of zooxanthellae. Compared with the well-studied zooxanthellae Symbiodiniaceae in coral holobionts, we rarely know about other coral symbiotic algae, let alone their thermal tolerance. In this study, a zoochlorellae, *Symbiochlorum hainanensis,* isolated from the coral *Porites lutea,* was proven to have a threshold temperature of 38°C. Meanwhile, unique high-temperature tolerance mechanisms were suggested by integrated transcriptomics and real-time quantitative PCR, physiological and biochemical analyses, and electron microscopy observation. Under heat stress, *S. hainanensis* shared some similar response strategies with zooxanthellae *Effrenium* sp., such as increased ascorbate peroxidase, glutathione peroxidase, superoxide dismutase activities and chlorophyll a, thiamine, and thiamine phosphate contents. In particular, more chloroplast internal layered structure, increased CAT activity, enhanced selenate reduction, and thylakoid assembly pathways were highlighted for *S. hainanensis*’s high-temperature tolerance. Notably, it is the first time to reveal a whole selenate reduction pathway from SeO_4_^2−^ to Se^2−^ and its contribution to the high-temperature tolerance of *S. hainanensis*. These unique mechanisms, including antioxidation and maintaining photosynthesis homeostasis, efficiently ensure the high-temperature tolerance of *S. hainanensis* than *Effrenium* sp. Compared with the thermosensitivity of coral symbiotic zooxanthellae Symbiodiniaceae, this study provides novel insights into the high-temperature tolerance mechanisms of coral symbiotic zoochlorellae *S. hainanensis*, which will contribute to corals’ survival in the warming oceans caused by global climate change.

**IMPORTANCE:**

The increasing ocean temperature above 31°C–32°C might trigger a breakdown of the coral-Symbiodiniaceae symbioses or coral bleaching because of the thermosensitivity of Symbiodiniaceae; therefore, the exploration of alternative coral symbiotic algae with high-temperature tolerance is important for the corals’ protection under warming oceans. This study proves that zoochlorellae *Symbiochlorum hainanensis* can tolerate 38°C, which is the highest temperature tolerance known for coral symbiotic algae to date, with unique high-temperature tolerance mechanisms. Particularly, for the first time, an internal selenium antioxidant mechanism of coral symbiotic *S. hainanensis* to high temperature was suggested.

## INTRODUCTION

Coral reef ecosystems, known as the rainforest of the oceans, are suffering a dramatic worldwide decline because of global climate change, e.g., ocean warming (1, 2). The well-studied coral symbiotic photosynthetic algae are dinoflagellates (Symbiodiniaceae, also known as zooxanthellae), which are generally sensitive to thermal stress with a lower threshold temperature ca. 31°C–32°C (3). It is for this reason the thermal response of coral symbiotic Symbiodiniaceae has been investigated (3–9). For instance, under elevated temperatures, chlorophyll synthesis in *Breviolum* sp. B1, *Cladocopium goreaui* C1, and *Durusdinium trenchii* D1a is upregulated (4), and the heat shock proteins (HSPs) display different expression changes among *Cladocopium* (clade C1) (5, 6), *Cladocopium* (clade C3K) (7), *Durusdinium* (clade D1) (5), *Durusdinium* (clade D2) (6), and *Fugacium* (clade F) (3). In addition, increased ascorbate peroxidase (APX) activity was detected in *Fugacium* (clade F1) at 33°C (8). Upregulated expression of genes encoding glutathione peroxidase (GPX), peroxiredoxin (Prdx), superoxide dismutase (SOD), and its different metalloforms are upregulated under heat stress (6, 9). These reports provide a preliminary understanding of the thermal response of coral symbiotic zooxanthellae Symbiodiniaceae and suggest that hosting high-temperature tolerant symbiotic algae is a valid strategy for corals to survive under higher temperatures (10).

The thermal tolerance of coral symbiotic algae is very important for the health and survival of coral holobionts in the warming oceans. Studies have found that some Symbiodiniaceae types have relatively higher thermal tolerance, e.g., *Symbiodinium thermophilum* (11) and *Durusdinium trenchii* (10, 12), but totally, coral symbiotic zooxanthellae Symbiodiniaceae is sensitive to thermal stress with lower threshold temperature, i.e., 31°C–32°C. The increasing ocean temperature above 32°C might trigger a breakdown of the coral-Symbiodiniaceae symbioses (13) or coral bleaching caused by thermosensitive Symbiodiniaceae’s escape or hypopigmentation (1, 2, 10, 14); therefore, the exploration of other kinds of coral symbiotic algae with high-temperature tolerance is important for the corals’ protection under warming oceans.

Besides zooxanthellae Symbiodiniaceae, corals host other kinds of symbiotic photosynthetic algal symbionts, e.g*., Ostreobium* (15). In 2018, a zoochlorellae, *Symbiochlorum hainanensis* (Chlorophyta, Ulvophyceae), was first isolated from the bleached scleractinian coral *Porites lutea* in the South China Sea and named by us (16). Meanwhile, we found that *S. hainanensis* was wildly distributed in scleractinian corals *Platygyra lamellina*, *Porites lutea,* and *Favia speciosa*. Particularly, the abundance of *S. hainanensis* became higher when these corals were bleached under thermal stress, accompanied by an abundant decrease in coral symbiotic Symbiodiniaceae (17). This phenomenon indicates the possible roles of *S. hainanensis* in maintaining the coral holobionts’ health under warming oceans by replacing zooxanthellae Symbiodiniaceae. Zoochlorellae has been found to be able to enhance the acclimation capacity of green hydra under heat stress (18), but we rarely know about the response of coral zoochlorellae to thermal stress compared to coral zooxanthellae. In 2020, we proved that *S. hainanensis* could maintain growth at 32°C, which is generally a lethal temperature to most Symbiodiniaceae (19). Thus, the high-temperature tolerance of zoochlorellae *S. hainanensis* arouses our great interest, and it is hypothesized that it probably has unique high-temperature tolerant mechanisms that are different from zooxanthellae Symbiodiniaceae. In this study, unique high-temperature tolerance mechanisms of coral symbiotic *Symbiochlorum hainanensis* to a high temperature of 38°C were predicted by transcriptomics first and then verified by real-time quantitative PCR (RT-qPCR) and physiological and biochemical analysis along with electron microscopy observation using thermosensitive zooxanthellae *Effrenium* sp. as a control.

## RESULTS

### Physiological and biochemical changes in *S. hainanensis* under thermal stress

**Fig 1:**
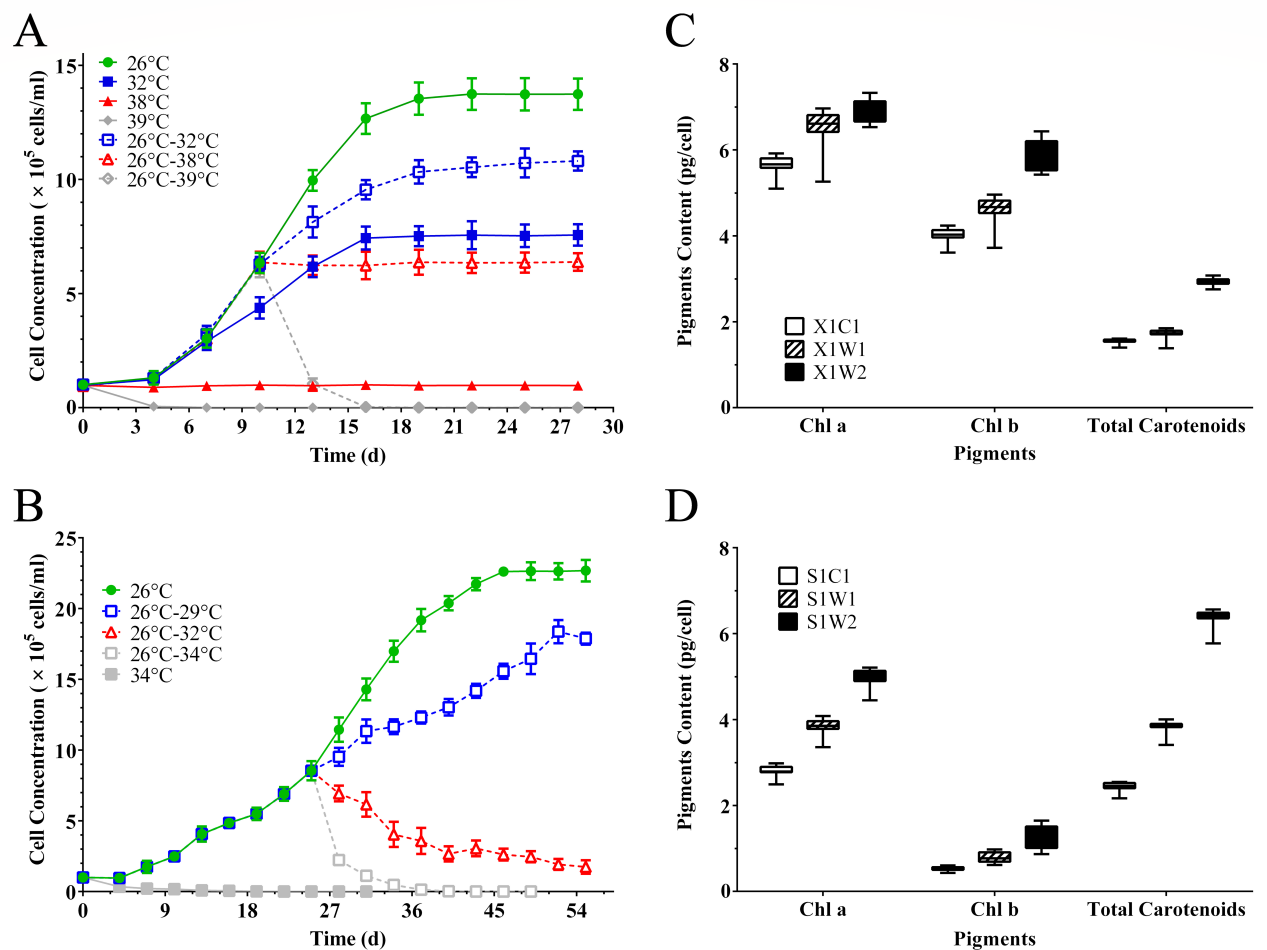
Growth curve and pigments’ content of *S. hainanensis* X1 (A and **C**) and *Effrenium* sp. S1 (**B and D**) under different temperatures. In panels A and B, error bars represent ±standard deviations (SD), and some error bars are obscured by data point markers; the batch experiments were conducted in triplicates. One of the representative data sets is presented here. In panels C and D, whiskers represent the minimum and maximum of at least five samples in three independent experiments. X1C1: 26°C; X1W1: 32°C; X1W2: 38°C; S1C1: 26°C; S1W1: 29°C; and S1W2: 32°C.

Zoochlorellae *S. hainanensis* grew well under 26°C and survived under 38°C but died when it was exposed to 39°C (Fig. 1A), demonstrating that 38°C is an extreme thermal stress to this alga. Contrary to *S. hainanensis*, zooxanthellae *Effrenium* sp. died quickly when it was exposed to 34°C (Fig. 1B), showing a lower heat tolerance than *S. hainanensis*.

**Fig 2:**
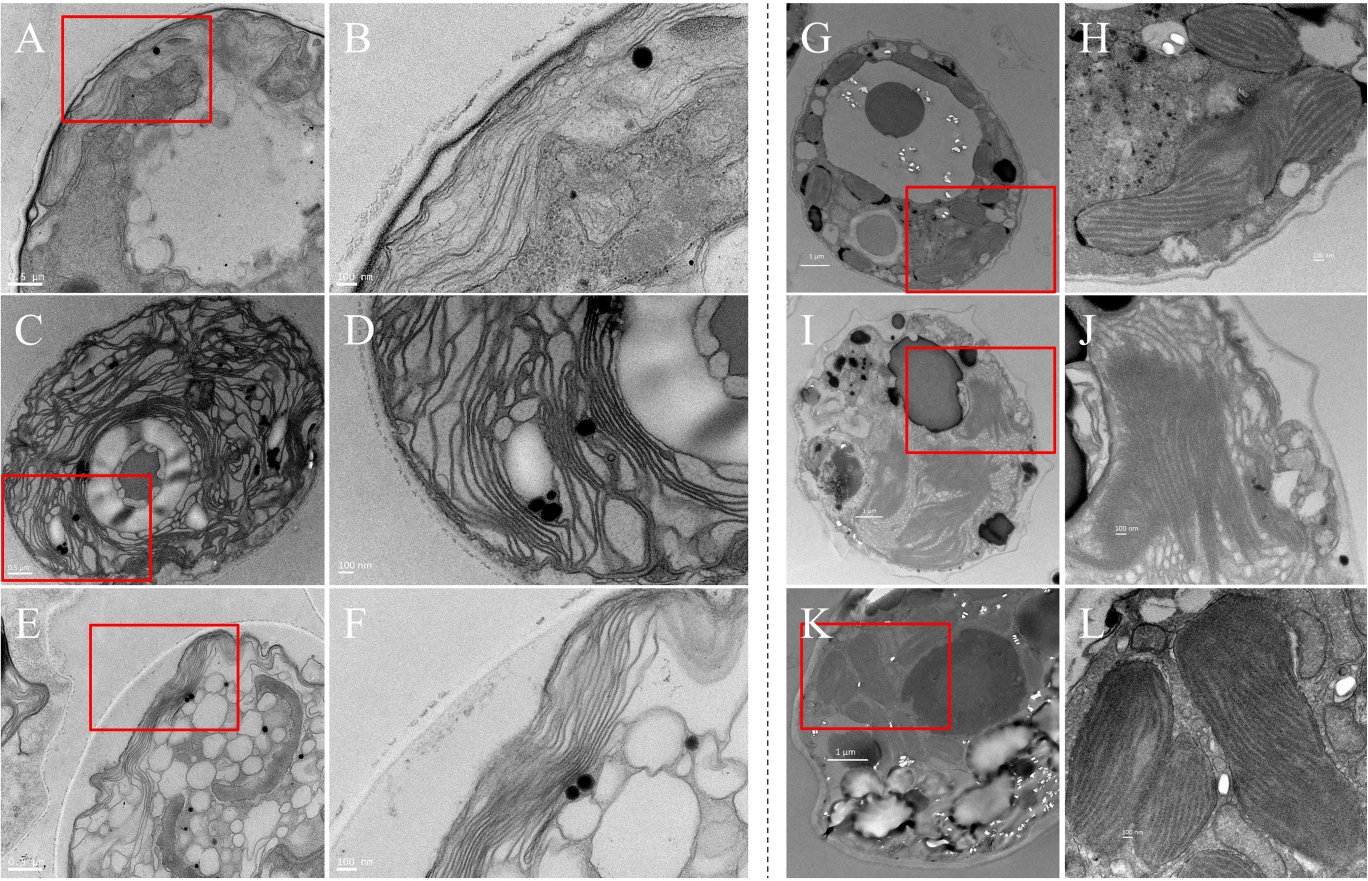
Transmission electron microscope images of *S. hainanensis* X1 and *Effrenium* sp. S1 under different temperatures. *S. hainanensis* X1 under the control (A, 26°C) and elevated temperatures (C, 32°C and E, 38°C). Panels B, D, and F are enlarged views of the parts of panels A, C, and E marked in red squares, respectively. *Effrenium* sp. S1 under the control (G, 26°C) and elevated temperatures (I, 29°C and K, 32°C). Panels H, J, and L are enlarged views of the parts of panels G, I, and K marked in red squares, respectively.

The content of chlorophyll a in *S. hainanensis* cells increased significantly under higher temperatures of 32°C and 38°C, compared with the 26°C control (Fig. 1C). This phenomenon also occurred in *Effrenium* sp. at 29°C and 32°C compared with the control at 26°C (Fig. 1D). The diameter of *S. hainanensis* cells was about 5–10 µm (Fig. 2A, C, and E). Interestingly, the internal layers of chloroplasts in *S. hainanensis* cells under higher temperatures (Fig. 2D and F) become more abundant compared with the control (Fig. 2B). In the case of *Effrenium* sp. (Fig. 2G through L), the diameter of cells was about 4–7 µm. However, there was no significant change in the internal layers in *Effrenium* sp. chloroplasts under heat stress, which was different from *S. hainanensis*.

**Fig 3:**
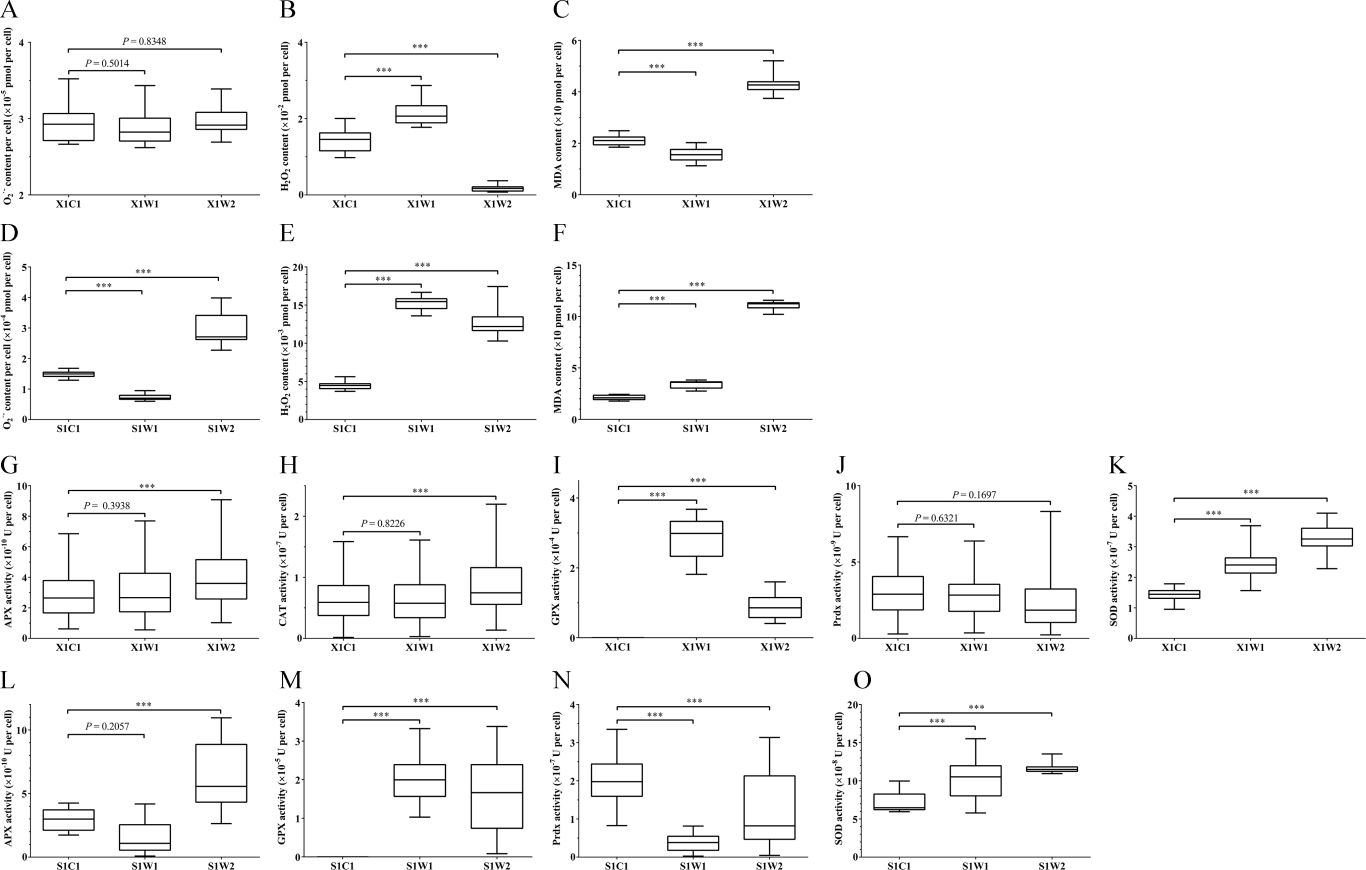
Physiological and biochemical analyses of *S. hainanensis* X1 and of *Effrenium* sp. S1 under different temperatures. Box plot of concentration of O_2_^•−^ anion per cell in *S. hainanensis* X1 (**A**) and *Effrenium* sp. S1 (**D**). Box plot of concentration of H_2_O_2_ per cell in *S. hainanensis* X1 (**B**) and *Effrenium* sp. S1 (**E**). Box plot of concentration of MDA per cell in *S. hainanensis* X1 (**C**) and *Effrenium* sp. S1 (**F**). Box plot of the activity of APX (**G**), CAT (**H**), GPX (**I**), Prdx (**J**), and SOD (**K**) per cell in *S. hainanensis* X1. Box plot of the activity of APX (**L**), GPX (**M**), Prdx (**N**), and SOD (**O**) per cell in *Effrenium* sp. S1. Whiskers represent the minimum and maximum of at least 15 samples in three independent experiments (at least five each). The statistical difference (one-way ANOVA) between treatment and control is indicated as *P < 0.05, **P < 0.01, or ***P < 0.001. X1C1: 26°C; X1W1: 32°C; X1W2: 38°C; S1C1: 26°C; S1W1: 29°C; and S1W2: 32°C. Some box plots are obscured by the *x* axis in panels **I** and **M**.

Based on this study, the O_2_^•−^ content in *S. hainanensis* did not change significantly under higher temperatures compared with the control (Fig. 3A). H_2_O_2_ content decreased significantly under the extreme high temperature of 38°C (Fig. 3B), whereas malondialdehyde (MDA) content increased significantly under the extreme high temperature of 38°C (Fig. 3C). In contrast, the O_2_^•−^, H_2_O_2_, and MDA contents of *Effrenium* sp. increased under higher temperatures (Fig. 3D through F).

Compared with the control at 26°C, the activities of antioxidases APX, CAT, GPX, and SOD in *S. hainanensis* increased particularly under the extremely high temperature of 38°C (Fig. 3G through I), whereas the Prdx activity remained the same as the control (Fig. 3J). In the case of *Effrenium* sp., the activities of antioxidases APX, GPX, and SOD displayed similar change trends as *S. hainanensis* (Fig. 3L through N). It is worth mentioning that, different from *S. hainanensis*, no significant CAT activity was detected in *Effrenium* sp., and the Prdx activity decreased in heat stress groups (Fig. 3O).

### Transcriptome profiles of *S. hainanensis* and *Effrenium* sp. under thermal stress

**Fig 4:**
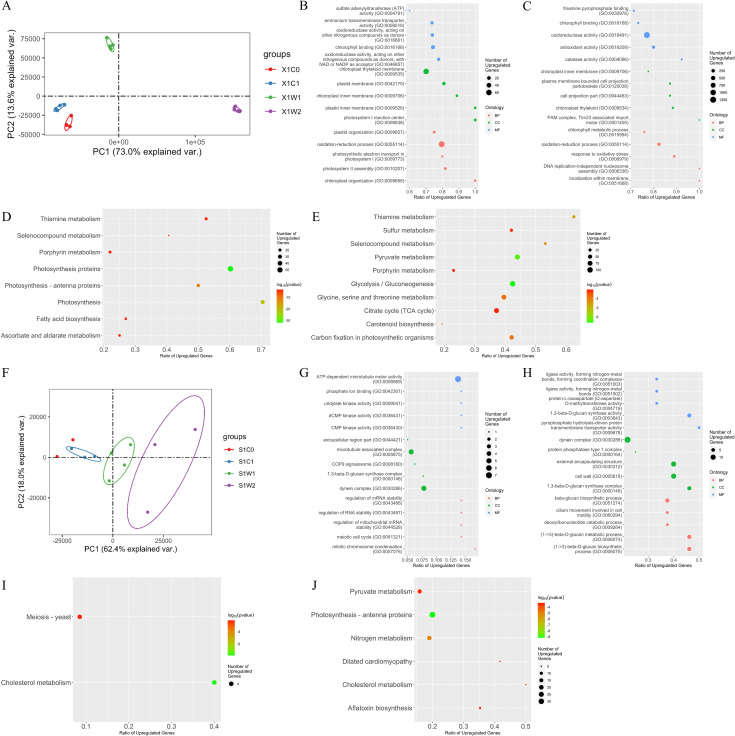
Visualization of PCA, KEGG enrichment, and GO enrichment analysis based on transcriptome. Plots of PCA of *S. hainanensis* X1 (**A**) and *Effrenium* sp. S1 (**F**) transcriptome profiles. Visualization of KEGG enrichment results of DEGs (*P*_adj_ < 0.01) in X1W1 group (**B**), X1W2 group (**C**), S1W1 group (**G**), and S1W2 group (**H**) (*P* value < 0.05). Visualization of top five terms of three ontologies in GO enrichment results of DEGs in X1W1 group (**D**), X1W2 group (**E**), S1W1 group (**I**), and S1W2 group (**J**). Treatments of each group are as follows: X1C0 group (26°C, 0 day, control), X1C1 group (26°C, third day), X1W1 group (32°C, third day), X1W2 group (38°C, third day); S1C0 group (26°C, 0 day, control), S1C1 group (26°C, third day), S1W1 group (29°C, third day), and S1W2 group (32°C, third day); quadruplicate in each group. For both KEGG enrichment and GO enrichment analysis, DEG-eliminated sequences from the X1C1 group or the S1C1 group were used for each alga, respectively. For panels **B**, **C**, **G,** and **H**, the *x* axis represents the ratio between the gene number of significantly upregulated genes and the gene number of genes annotated in each pathway; size of the burbles represents the number of genes that showed significantly different expression; color of the burbles represents log_10_(*P*-value). For panels **D**, **E**, **I,** and **J**, terms were sorted by the ratio of upregulated DEGs in total DEGs in reverse order; *x* axis represents the ratio between the gene number of significantly upregulated genes and the gene number of genes annotated in each term; size of the burbles represents the number of genes that showed significant upregulation; color of the burbles represents different ontology.

**Fig 5:**
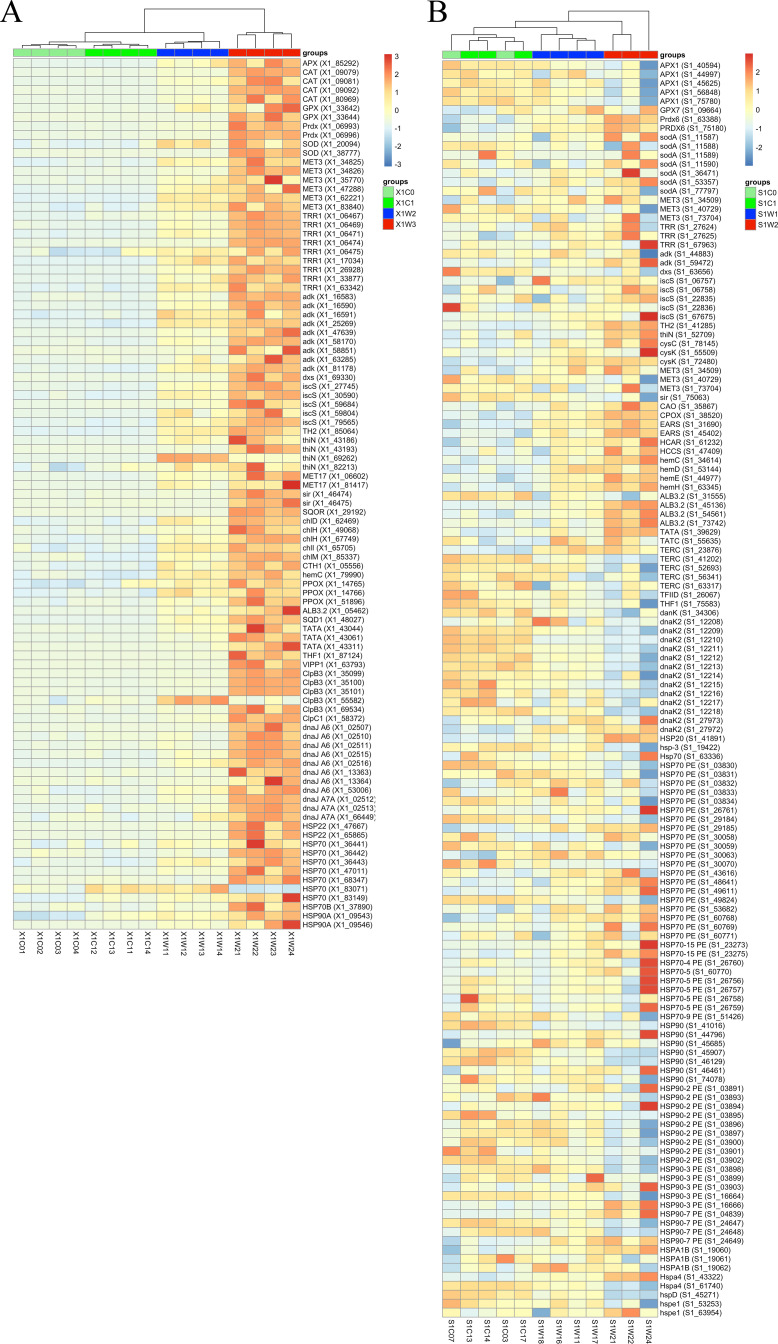
Heatmap of DEGs in *S. hainanensis* X1 (**A**) and same function genes in *Effrenium* sp. S1 (**B**). DEGs (*P*_adj_ <0.01) of *S. hainanensis* X1 exposed to thermal stress (32°C and 38°C; *n* = 4) and same function genes from *Effrenium* sp. S1 exposed to thermal stress (29°C and 32°C; *n* = 4). Genes related to antioxidation, selenate reduction, thiamine biosynthesis, pigments’ synthesis, thylakoid formation, chloroplast chaperone, and HSPs are shown. X1C0: 26°C, 0 day; X1C1: 26°C, the third day; X1W1: 32°C; X1W2: 38°C; S1C0: 26°C, 0 day; S1C1: 26°C, the third day; S1W1: 29°C; and S1W2: 32°C.

The RNA sequencing (RNA-Seq) data and *de novo*-assembled unigenes of *S. hainanensis* and *Effrenium* sp. are summarized in Tables S1 and S2, respectively, and differentially expressed genes (DEGs) of both algae are listed in Tables S3 and S4, respectively. The principal component analysis (PCA) showed that the gene transcriptions of *S. hainanensis* were obviously different between the control and thermal stress groups (Fig. 4A). The top five terms of three GO ontologies were related to photosynthesis and antioxidation (Fig. 4B through C). In particular, selenocompound metabolism (ko00450) and thiamine metabolism (ko00730) were significantly changed under higher temperatures (Fig. 4D and E), indicating their possible relationship with thermal tolerance. The expression of two key genes (*MET3* and *TRR1*) in selenocompound metabolism was significantly upregulated (Fig. 5A). Five key genes (*adk*, *dxs, iscS*, *TH2,* and *thiN*) related to thiamine metabolism and two genes (*MET17* and *sir*) in sulfur metabolism (ko00920) exhibited significantly upregulated expression (Fig. 5A).

In addition, the expression of five genes (*ALB3.2*, *SQD1*, *TatA*, *Thf1,* and *VIPP1*) associated with thylakoid formation and seven genes (*ChlD*, *ChlH*, *ChlI*, *CHLM*, *CTH1*, *HEMC,* and *PPOX*) in chlorophyll a biosynthesis pathway was upregulated (Fig. 5A). In *S. hainanensis*, a large number of genes encoding HSPs and molecular chaperones in chloroplast were upregulated under heat stress (Fig. 5A). Genes encoding antioxidant enzymes (APX, CAT, GPX, and SOD) were significantly upregulated in *S. hainanensis* (Fig. 5A), which was consistent with increased activities (Fig. 3).

PCA, GO enrichment, and KEGG enrichment analyses were also performed for *Effrenium* sp. (Fig. 4F through J). In order to compare the heat resistance mechanisms of these two different algae, only the genes in *Effrenium* sp. corresponding to the DEGs in *S. hainanensis* were compared, regardless of the level of change in gene expression. Much less DEGs were detected in *Effrenium* sp. (8,543 DEGs, Table S4) than in *S. hainanensis* (29,393 DEGs, Table S3). The top GO terms were related to the cell cycle, and only one KEGG pathway (i.e., cholesterol metabolism) exhibited significant changes in both thermal stress groups. In *Effrenium* sp., the expression of nine genes in chlorophyll a biosynthesis was upregulated (Fig. 5B), which is similar to *S. hainanensis*. Similar to *S. hainanensis*, most genes related to thiamine metabolism in *Effrenium* sp. were upregulated under heat stress (Fig. 5B). But totally, *Effrenium* sp. showed different transcriptome profiles from *S. hainanensis*; for example, only a part of HSP transcripts (mainly *HSP70*) was upregulated in *Effrenium* sp. under heat stress (Fig. 5B), and no CAT-encoding transcript change was detected in *Effrenium* sp. Meanwhile, the upregulated *TRR1* transcripts were not significant under higher temperatures; some *MET3* transcripts were upregulated, while others were downregulated. In sulfur metabolism, one *sir* transcript was downregulated and *MET17* was not detected. In the case of genes associated with thylakoid formation, only *ALB 3.2* and *TatA* transcripts and one of the five thylakoid membrane protein TERC-encoding transcripts were upregulated, while others (such as *TatC*, *THFIID,* and *THF1*) displayed a decreasing trend.

### Validation of RNA-Seq-based DEGs by RT-qPCR and compound analyses

**Fig 6:**
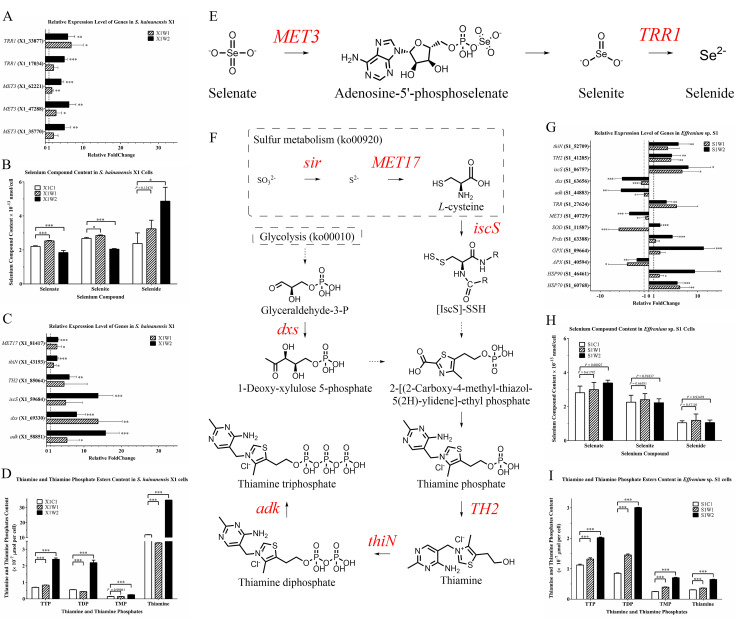
RT-qPCR verification and selenate reduction, thiamine, and thiamine phosphate compounds’ analyses. Bar plot of RT-qPCR results (**A**) and selenium compounds’ content (**B**) in the selenate reduction pathway in *S. hainanensis* X1. Bar plot of RT-qPCR results (**C**) and thiamine and thiamine phosphates’ content (**D**) in the thiamine biosynthesis pathway in *S. hainanensis* X1. Scheme of the detected upregulated selenate reduction pathway (**E**) and upregulated thiamine biosynthesis pathway (**F**) in *S. hainanensis* X1. Bar plot of RT-qPCR results (**G**), selenium compounds’ content (**H**), and thiamine and thiamine phosphates’ content (**I**) in *Effrenium* sp. S1. For RT-qPCR, the data represent mean ± SD of quadruplicates; β-actin (X1_44387) and 18S rRNA (S1_42608) were used as internal controls, respectively. For compound contents’ results, the data represent mean ± SD of at least six samples in two independent experiments (at least three each). The statistical difference (one-way ANOVA) between treatment and control is indicated as *P < 0.05, **P < 0.01, or ***P < 0.001. For the pathway, solid and dotted arrows represent one-step or multi-step reactions, respectively; italics represent genes; the color indicates the relative expression change (red represents upregulated). X1C1: 26°C; X1W1: 32°C; X1W2: 38°C; S1C1: 26°C; S1W1: 29°C; and S1W2: 32°C.

In *S. hainanensis*, 17 DEGs based on RNA-Seq-analysis exhibited upregulated expression in the RT-qPCR analysis, which confirmed the RNA-Seq-based results. In the selenate reduction pathway (Fig. 6A), transcripts of *MET3* and *TRR1* transcripts were upregulated in both the 32°C (1.74–6.12-fold) and 38°C (4.17–6.09-fold) groups. In the thiamine biosynthesis pathway (Fig. 6C), transcripts of *adk*, *dxs*, *iscS*, *TH2*, *thiN,* and *MET17* were upregulated by 1.79–12.28-fold in the 32°C group and 2.80–15.42-fold in the 38°C group, respectively. Transcripts of *CAT*, *GPX*, *SOD*, *HSP22*, *HSP70,* and *HSP90* were upregulated by 1.96–12.05-fold in the 32°C group and 2.71–26.39-fold in the 38°C group (Table S5), respectively. Based on the RT-qPCR analysis of *Effrenium* sp., 13 DEGs (Table S6) exhibited the same change trend with RNA-Seq results. Transcripts of *MET3*, *adk*, *dxs,* and *APX* were downregulated in both the 29°C (1.37–3.10-fold) and 32°C (2.07–5.29-fold) groups. Transcripts of *TRR*, *iscS*, *TH2*, *thiN*, *GPX*, *Prdx*, *HSP70,* and *HSP90* were upregulated in both the 29°C (1.38–6.32-fold) and 32°C (4.35–11.03-fold) groups, and the transcript of *SOD* was downregulated by 5.90-fold in the 29°C group and upregulated by 2.26-fold in the 32°C group.

In order to verify the results from DEGs and RT-qPCR analyses, SeO_4_^2−^ , SeO_3_^2−^, and Se^2−^ contents in *S. hainanensis* and *Effrenium* sp. cells were analyzed. In *S. hainanensis* (Fig. 6B), when compared with the 26°C control, Se^2−^ content increased in the 32°C group (36.3%, *P* = 0.13) and significantly increased in the 38°C group (104.6%, *P* < 0.001); both SeO_4_^2−^ and SeO_3_^2−^ contents decreased significantly in the 38°C group (SeO_4_^2−^, 23.5% and SeO_3_^2−^, 16.4%). In contrast, the contents of SeO_4_^2−^, SeO_3_^2−^, and Se^2−^ in *Effrenium* sp. showed no significant change in the 29°C group (*P* > 0.5) and 32°C group (*P* > 0.05), when compared with the 26°C control (Fig. 6H).

The thiamine and thiamine phosphates’ contents in *S. hainanensis* and *Effrenium* sp. cells were also analyzed. In *S. hainanensis* (Fig. 6D), the contents of thiamine, thiamine monophosphate (TMP), thiamine diphosphate (TDP), and thiamine triphosphate (TTP) changed under higher temperatures when compared with the control. In the 32°C group, TTP content increased significantly (19.9%), while TMP content exhibited no significant change (*P* = 0.99), thiamine content and TDP content decreased significantly (thiamine, 71.3% and TDP, 20.8%). Especially under the extremely high temperature of 38°C, thiamine, TMP, TDP, and TTP increased by 195.5%, 73.5%, 296.7%, and 243.2%, respectively. In the case of *Effrenium* sp. (Fig. 6I), the contents of thiamine, TMP, TDP, and TTP increased in the 29°C group (thiamine, 18.8%; TMP, 59.2%; TDP, 70.9%; and TTP, 17.4%) and the 32°C group (thiamine, 112.4%; TMP, 185.3%; TDP, 254.6%; and TTP, 80.0%), respectively, compared with the 26°C control.

## DISCUSSION

**Fig 7:**
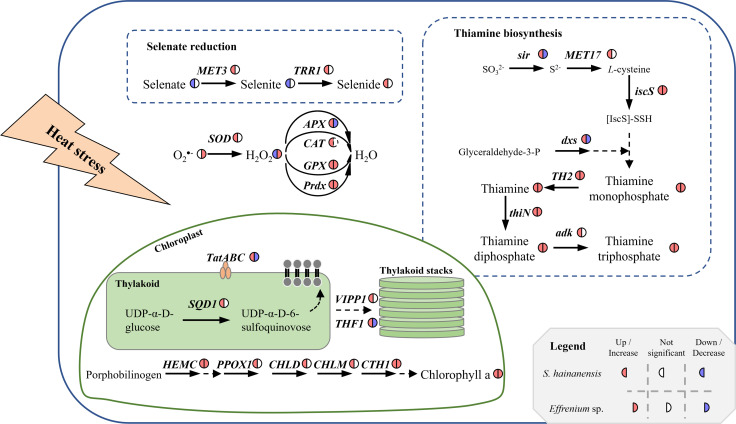
Schematic summary of the upregulated DEGs in *S. hainanensis* X1 response to thermal stress. DEGs were selected as adjusted *P* value < 0.01 and log2 (fold change) ≥ ±1 in thermal stressed groups (X1W1 group, 32°C and X1W2 group, 38°C) compared to the control (X1C1 group, 26°C) on the third day. Genes related to antioxidases, selenate reduction, thiamine biosynthesis, chlorophyll a synthesis, and thylakoid formation are shown. Solid and dotted arrows represent one-step or multi-step reactions, respectively. Italics represent genes. The semicircle indicates the change of gene relative expression or substance content in algae cells: left semicircle represents *S. hainanensis* X1, and right semicircle represents *Effrenium* sp. S1; color in the semicircle indicates the change trend: red represents upregulated or increased, blue represents downregulated or decreased, and white represents no significant chang. The dotted black border represents that this gene or substance was not detected.

Based on the thermal response of coral Symbiodiniaceae (3, 5–9) and *Effrenium* sp., in this study, *S. hainanensis* has the highest temperature tolerance known for coral symbiotic algae, i.e., 38°C. The expression of HSPs is commonly considered to be associated with stress (20), HSP-related DEGs in zoochlorellae *S. hainanensis* and zooxanthellae *Effrenium* sp. under thermal stress indicate that algal cells were indeed in a stress response state (Fig. 5). HSPs are known to have function in protein processing, such as protein folding, protein translocation, and maintaining the conformation of unstable/wrong-folded proteins, as well as their signaling functions (20). In *S. hainanensis*, the upregulation of genes encoding HSPs, especially small HSPs, HSP70 and HSP90, indicated that the heat response system was triggered, and the alga was conserving its protein homeostasis. In contrast to *S. hainanensis*, a few HSPs were upregulated in *Effrenium* sp., indicating that some of its protein homeostasis maintaining mechanism might be damaged or dysfunctional under thermal stress, which would result in the destruction of protein homeostasis inside algal cells. However, the unchanged O_2_^•−^ content and decreased H_2_O_2_ content in *S. hainanensis* indicated its higher ability to remove reactive oxygen species (ROS) than *Effrenium* sp. (Fig. 3A through F); this is probably one of the reasons why *S. hainanensis* can survive under the extreme high temperature of 38°C. Compared with *Effrenium* sp., the presence of CAT, the remaining activity of Prdx, and the reducing product of the selenate reduction pathway (Se^2−^) could be the mechanisms that contribute to the enhanced ROS removal capacity in *S. hainanensis*. The upregulated DEGs related to antioxidases, selenate reduction, thiamine biosynthesis, chlorophyll a synthesis, and thylakoid assembly in *S. hainanensis* X1 are summarized in Fig. 7, showing the unique thermal resistance mechanisms of *S. hainanensis*. Considering the wide distribution and increased abundance of *S. hainanensis* in bleaching corals (16, 17, 19, 21), *S. hainanensis* might play important roles in corals’ resistance to thermal stress, particularly when thermosensitive zooxanthellae escape in the warming oceans caused by global climate change. Meanwhile, the transplanting of *S. hainanensis* might be a strategy to help corals survive in warming oceans caused by global climate change because of its high thermal tolerance.

### The specific contribution of antioxidant enzymes, particularly CAT, to the high-temperature tolerance of *S. hainanensis*

Elevated temperature not only affects temperature-dependent biochemical reactions but also increases intracellular oxidative pressure (22). In this study, the MDA content change may reflect the oxidative pressure particularly under the ultimate temperatures, i.e., 38°C and 32°C, for *S. hainanensis* and *Effrenium* sp., respectively (Fig. 3C and F). The increase in ROS such as O_2_^•−^ and H_2_O_2_ under heat stress will cause algal oxidative damage (22). Hence, the scavenging of ROS will contribute to heat resistance. The O_2_^•−^ content in *S. hainanensis* under different experiment temperatures remained low, and the H_2_O_2_ content under the extreme temperature significantly decreased (Fig. 3A and B). On the contrary, O_2_^•−^ and H_2_O_2_ contents in *Effrenium* sp. increased (Fig. 3D and E), indicating that its ROS removal capacity was weakened under thermal stress. The lower O_2_^•−^ and H_2_O_2_ contents in *S. hainanensis* indicate its higher ability to relieve oxidative pressure than *Effrenium* sp.

The expression of genes encoding antioxidant enzymes SOD, APX, CAT, and GPX in *S. hainanensis* was upregulated under thermal stress (Fig. 5A); consequently, the activities of SOD, APX, CAT, and GPX increased (Fig. 3G through K). It is known that antioxidases are able to transfer O_2_^•−^ to H_2_O_2_ and finally to H_2_O (22); therefore, ROS inside the algal cells is maintained at a low level. However, the different activity changes of these four enzymes indicated their different contributions to the heat resistance of *S. hainanensis*. Specifically, the GPX and SOD activities were higher when the algal cells were heated (32°C and 38°C), but the APX and CAT activities were only higher under an extremely high temperature (38°C). It can be speculated that GPX and SOD are the core antioxidant enzymes in the heat resistance of *S. hainanensis*, and APX and CAT are reserves, which can only be called under the extremely high temperature. The increased intercellular SOD activity in *Chlorella ellopsoidea* (23), *Breviolum* (clade B1), and *Cladocopium* (clade C1) (8) indicates SOD’s role in the algal response to heat stress. In *Chlamydomonas reinhardtii*, APX activity was found to be increased under elevated temperatures (24). Although transcripts encoding antioxidant enzymes like APX and SOD displayed a decreasing trend in *Effrenium* sp. under thermal stress, their expression changes did not reach a significant level. Although catalase peroxidase (KatG) has been found in *Breviolum* (clade B1) (25), the capacity of H_2_O_2_ degradation in *Breviolum* (clade B1) and *Effrenium* (clade E1) displayed no significant change under thermal stress (25, 26). Accordingly, it is speculated that different coral symbiotic algae have different response patterns of antioxidant enzymes to thermal stress. In particular, our results suggested the importance of antioxidase CAT in the high-temperature tolerance of *S. hainanensis* because no CAT activity was detected in *Effrenium* sp. Based on the result from Bayer et al. (27), *Symbiodinium* sp. CassKB8 and *Breviolum* sp. Mf1.05b appear to lack CAT (23), and no CAT activity change was detected in dinoflagellate *Cladocopium goreaui* during the thermal exposure period (28). Thus, CAT might lead to a much more effective antioxidant system in *S. hainanensis* and aid its higher tolerance than *Effrenium* sp.

### Selenate reduction and thiamine biosynthesis related to the high-temperature tolerance of *S. hainanensis*

In *S. hainanensis*, besides the roles of multiple antioxidant enzymes, the enhancement of selenate reduction and thiamine biosynthesis pathways probably contributes to the tolerance of *S. hainanensis* to high temperatures (Fig. 7). It is worth mentioning that a whole pathway of selenate reduction was detected in *S. hainanensis* (Fig. 6E), which was correlated with this algal high-temperature tolerance. In this pathway, SeO_4_^2−^ is successively catalyzed to Se^2−^ by sulfate adenylyltransferase (EC 2.7.7.4, encoded by *MET3*) and thioredoxin reductase (EC 1.8.1.9, encoded by *TRR1*). The upregulated expression of two genes *MET3* and *TRR1* (Fig. 5A and 6A) and the increased content of Se^2−^ in *S. hainanensis* cells (Fig. 6B) were detected under heat stress in this study. As a result of this upregulation, theoretically, the content of substrate (SeO_4_^2−^) and intermediate (SeO_3_^2−^) should be reduced, which was supported by the decreased contents of SeO_4_^2−^ and SeO_3_^2−^ and the increase of Se^2−^ in the algal cells. In contrast to *S. hainanensis*, there is no significant content change in SeO_3_^2−^ or Se^2−^ in *Effrenium* sp. cells (Fig. 6H), as well as no significant change in the related genes’ expression (Fig. 5B), indicating this selenate reduction pathway does not contribute to the heat resistance of *Effrenium* sp. Selenium has been reported to play a key role in the cellular antioxidant defense mechanism (29). For example, Se at low concentration positively promoted the antioxidative effect of *Chlorella pyrenoidosa* by increasing the levels of glutathione peroxidase, catalase, linolenic acid, and photosynthetic pigments (30) and increased the activity of antioxidant enzymes (SOD and CAT) and the amount of antioxidant metabolites (phenols, flavonoids, and carotenoids) in *Ulva* sp. (31). Maronić et al. (32) also highlighted the importance of the algal Se detoxification strategy, especially the role of selenoenzymes and other selenoproteins with antioxidant function. Similarly, based on the upregulation of specific genes and the increased Se^2−^ yield concentration under heat stress, this study suggests an internal selenium antioxidant mechanism of *S. hainanensis* to high temperature. Taken together the present knowledge of the thermal response mechanisms of well-studied *Chlamydomonas* (33), coral symbiotic Symbiodiniaceae (3–9), and the thermal response of *Effrenium* sp. in this study, it is the first time to find a correlation between the upregulated selenate reduction pathway and high-temperature tolerance of coral symbiotic algae, which could be one of the reasons why its antioxidant system is more effective than *Effrenium* sp.

Tunc-Ozdemir et al. (34) found the role of thiamine in the protection of cells against oxidative damage in *Arabidopsis thaliana* and found that thiamine-induced tolerance to oxidative stress was accompanied by decreased production of reactive oxygen species, as evidenced from decreased protein carbonylation and hydrogen peroxide accumulation. In this study, the expression of six genes (*MET17*, *iscS*, *dxs*, *TH2*, *thiN,* and *adk*) responsible for synthesizing thiamine and its three phosphates was upregulated in *S. hainanensis* under heat stress (Fig. 5A, 6C, D, and F), which was proved by the increased contents of thiamine, TMP, TDP, and TTP under heat stress. Prior studies have noted that the antioxidant/anti-heat function of thiamine and TDP is common in algae and plants, such as the cyanobacterium *Nodularia spumigena*, dinoflagellate *Prorocentrum minimum* (35), *Zea mays,* and *Arabidopsis thaliana* (36). Thus, thiamine biosynthesis could contribute to thermal tolerance in *S. hainanensis* by increasing the content of its antioxidant products thiamine and TDP. Combined with the similar increase of thiamine and TDP in thermally stressed *Effrenium* sp., it could be proposed that this is a universal mechanism in the thermal stress response of coral symbiotic algae. The increased TTP of algae under thermal stress (Fig. 6D) suggests TTP’s possible correlation with thermal tolerance. However, to date, we rarely know about the function of TTP in stress response, except that it was suggested to serve as “alarmones” when cells are under starvation (37). Thus, TTP’s roles in the antioxidation of *S. hainanensis* need further study.

### Maintenance of photosynthesis homeostasis by enhancing thylakoid assembly for the high-temperature tolerance of *S. hainanensis*

Based on the KEGG and GO enrichment analyses, in addition to antioxidation, photosynthesis homeostasis maintenance might be another contributor to the high-temperature tolerance of *S. hainanensis*. Under heat stress, the expression of seven genes (*ChlD*, *ChlH*, *ChlI*, *CHLM*, *CTH1*, *HEMC,* and *PPOX*) involved in chlorophyll a biosynthesis was upregulated (Fig. 5A). Consistent with this result, the chlorophyll a content in *S. hainanensis* cells increased (Fig. 1C). The enhanced chlorophyll a biosynthesis indicated that *S. hainanensis* was compensating for heat-induced chlorophyll loss or increasing the energy inflow under heat stress. In addition, the increased thylakoid formation was found to be involved in the response of *S. hainanensis* to heat stress (Fig. 7). Five genes (*ALB3.2*, *SQD1*, *TatA*, *Thf1,* and *VIPP1*) associated with thylakoid formation were upregulated in *S. hainanensis* under heat stress (Fig. 5A). These genes are involved in thylakoid membrane lipid synthesis (*SQD1*), thylakoid membrane protein synthesis (*TatA*), integration of light-harvesting complex into thylakoid membrane (*ALB3.2*), as well as thylakoid assembly and stacking (*Thf1* and *VIPP1*). In contrast, the increased thylakoid assembly was not observed in *Effrenium* sp. (Fig. 2G through L, Fig. 5B), indicating the possible damage to photosynthesis homeostasis of this alga under heat stress. The internal layers of chloroplasts in *S. hainanensis* cells under higher temperatures (Fig. 2D and F) became more abundant compared with the control (Fig. 2B), whereas there was no significant change in internal layers in *Effrenium* sp. chloroplasts under heat stress. Thus, it can be speculated that *S. hainanensis* probably increase the assembly or the formation of thylakoids under thermal stress (Fig. 7). Presumably, it might be compensating for the thylakoid losses caused by heat or forming new thylakoid *de novo* to maintain photosynthesis energy inflow in *S. hainanensis*. Similar to our results, the formation of aberrant prolamellar body-like structures was observed in the chloroplast of heat-tolerating *Chlamydomonas reinhardtii* under elevated temperature, which is considered to be associated with photosynthesis maintenance (38). Similarly, the enlargement of chloroplasts along with the increase in chlorophyll fluorescence and pigment content of *S. hainanensis* were detected in our previous study (19). Coupled with the morphologic change of chloroplasts in both the 32°C and 38°C groups (Fig. 2), it is presumably suggested that *S. hainanensis* probably try to maintain the photosynthesis homeostasis by increasing the assembly of thylakoid and more chloroplast internal layered structure.

### Conclusions

Compared with the thermosensitive zooxanthellae *Effrenium* sp. (threshold temperature: 32°C), zoochlorellae *S. hainanensis* has a heat-resistant temperature of 38°C, which is the highest thermal tolerance of coral symbiotic algae. Besides the similar heat response strategies as *Effrenium* sp., e.g., increased APX, GPX, and SOD activities and chlorophyll a, thiamine, and thiamine phosphates’ contents, *S. hainanensis* has unique high-temperature tolerance mechanisms, including more chloroplast internal layered structure, increased CAT activity, and enhanced selenate reduction and thylakoid assembly pathways. Particularly, for the first time, an internal selenium antioxidant mechanism of coral symbiotic *S. hainanensis* to high temperature was suggested. The revealed unique high-temperature tolerance mechanisms of zoochlorellae *S. hainanensis* efficiently remove ROS to maintain the low-level inner cellular superoxide (O_2_^•−^) content and ensure photosynthesis homeostasis. The revealed 38°C high-temperature tolerance and the related molecular mechanisms of *S. hainanensis* greatly expand our understanding of the heat resistance of coral symbiotic algae.

## MATERIALS AND METHODS

### *S. hainanensis* cultivation and thermal treatment

*S. hainanensis* X1 was isolated from bleached coral *Porites lutea* in the South China Sea and submitted to the China Center for Type Culture Collection (Wuhan, China) under the accession number CCTCC M2018096 (16). *Effrenium* sp. S1 was isolated from coral *Acropora hyacinthus* and *Galaxea fascicularis* in the South China Sea, Hainan, China (18°18ʹ52.8ʺ N, 109°46ʹ07.9ʺ E) by Professor Pengcheng Fu’s group and classified by us using the nuclear large subunit rDNA sequence (NCBI OR987794) as described by LaJeunusse et al. (39). Light incubator (PGX-80B, Tianling, Jiangsu, China) was used to culture algal seeds using Asp-8a medium (40) at 26°C, irradiance of 80–100 µmol quanta m^−2^ s^−1^ with a 12-/12-h light/dark cycle (19).

To determine the extreme thermal stress temperature, the cultures of *S. hainanensis* under 26°C were changed to 32°C, 38°C, and 39°C, respectively, on the 10th day (mid-exponential phase), while the cultures of *Effrenium* sp. under 26°C were changed to 29°C, 32°C, and 34°C, respectively, on the 25th day (mid-exponential phase). The initial cell density was 1 × 10^5^ cell/mL, and five replicate cultures were used. From the fourth day, algal cells were sampled by sterile dropper every 3 days and then stained to distinguish the viable cells as described by Malerba et al. (41). The number of viable cells was counted using a light microscope, with five replicate counts performed.

To reveal the thermal tolerance mechanisms of *S. hainanensis* and *Effrenium* sp., both algae were exposed to 32°C and 38°C, 29°C and 32°C, respectively, using 26°C as the control. The controls before and after the heat treatment (named C0 and C1) were used to rule out the effects of time (Table S7). The thermal treatment started on the 10th day for *S. hainanensis* and on the 25th day for *Effrenium* sp., i.e., their mid-exponential phase, and lasted for 3 days. Then, the algal cells (heated for 3 days and the control) were collected by centrifugation (6,500 × *g*, 15 min) using a high-speed refrigerated centrifuge (H1650R, Cence, Hunan, China), after being washed three times with sterile artificial seawater (NaCl, 453.80 mM; MgCl_2_·6H_2_O, 25.72 mM; CaCl_2_, 10.28 mM; MgSO_4_, 27.46 mM; KCl, 9.73 mM; NaHCO_3_, 2.40 mM; and NaBr, 0.81 mM). The collected algal cells were used for the following analyses.

### Pigments’ content analysis and transmission electron microscope observation

Pigments’ content was determined using the acetone-based method (42). Specimen sections with a thickness of about 70 nm were sliced using a cryo-ultramicrotome (UC6FC7, Leica, Wetzlar, Germany) and observed by a 120-kV transmission electron microscope (Tecnai G2 Spirit Bio twin, FEI Corp., Hillsboro, OR, USA) (17).

### Antioxidation biochemical analyses

Algal cells were ground in liquid nitrogen. The contents of superoxide (O_2_^•−^) and hydrogen peroxide (H_2_O_2_) were determined as described by Malerba and Cerana (43) and Gao et al. (44), respectively, to evaluate the intercellular ROS level. The level of membrane lipid peroxidation and the content of MDA were measured according to Malerba and Cerana (43). Five replicates were used for each test.

APX activity was detected using the kit D799461-0050 (Sangong, Shanghai, China). One unit of APX activity was defined as the amount of enzyme that oxidizes 1 µmol of ascorbate per minute in the reaction system. Catalase activity was detected by the kit D799597-0050 (Sangong, Shanghai, China). One unit of CAT activity was defined as the degradation of 1 µmol H_2_O_2_ per minute in the reaction system. GPX activity was detected by the kit D799617-0050 (Sangong, Shanghai, China). One unit of GPX activity was defined as the oxidation of 1 µmol NADPH per minute in the reaction system. SOD activity was detected by the kit D799593-0050 (Sangong, Shanghai, China). One unit of SOD activity was defined as the amount of enzyme that inhibited the rate of ferricytochrome *c* reduction by 50%. Prdx activity was determined by the kit D799592-0100 (Sangong, Shanghai, China). One unit of Prdx activity was defined as every 0.005 change of *A*_470_ per minute per milliliter in the reaction system.

### Transcriptome analysis

Total RNA was extracted as described in our previous study (19). The extracted RNA was divided into two parts, one for RNA sequencing and another for real-time quantitative PCR confirmatory analysis. In the case of RNA-Seq, 16 libraries (four replicates × four groups [C0, C1, W1, and W2] for each alga) were constructed and sequenced.

The quality control and short read assembly of RNA-Seq data were performed as described in our previous study (19). Transcripts with read counts ≤ 1 were discarded to reduce interference. The annotation of *de novo*-assembled unigenes was performed according to our previous study (19). Differentially expressed gene analysis was performed using DESeq2 R package version 1.26.0 (45), the thresholds to evaluate the significance for contigs were set as *P* value = 0.01 and log_2_ (fold change) = ±1. For differently expressed gene analysis, after eliminating DEGs from the C1 control group, GO enrichment and KEGG enrichment were performed using clusterProfiler v.3.14.3 R package (46). In GO enrichment, the default arguments were used; for each term, the proportion of upregulated DEGs in total DEGs was calculated and sorted in reverse order. In KEGG enrichment analysis, the default arguments were used, and the thresholds to evaluate the significance of change for each pathway were set as *P* value = 0.05.

### Real-time quantitative PCR analysis

A Tiangen FastKing RT Kit KR116 (Tiangen, Beijing, China) was used for the first-strand cDNA synthesis. RT-qPCR was conducted using Tiangen Talent qPCR PreMix (SYBR Green, FP209). The selected unigenes and primers (designed based on their sequence) are listed in Tables S5 and S6 for *S. hainanensis* and *Effrenium* sp., respectively. A BioRad C1000 Thermal Cycler (BioRad, Hercules, CA, USA) was used for PCR: 95°C for 5 min; 55°C for 20 s, and 72°C for 20 s, 40 cycles. The melting curve procedure was as follows: 95°C for 30 s; 55°C for 65 s and then rose to 95°C. The 2^−∆∆Ct^ method was used to calculate relative gene expression values (47).

### Detection of selenate and selenide in algal cells

The extraction of inorganic selenium was performed as described by Hartikainen et al. (48) with three replicates. The extracts were divided into two parts, one was used to detect the content of SeO_4_^2−^ and selenite (SeO_3_^2−^) immediately by high-performance liquid chromatography-inductively coupled plasma mass spectrometry (HPLC-ICPMS), which was labeled as the c(SeO_4_^2−^)_1_ and c(SeO_3_^2−^)_1_, respectively. A total of 0.2 g of K_2_S_2_O_8_ was added to another part of the extract and heated at 90°C for 1 h, and then the contents of SeO_4_^2−^ and SeO_3_^2−^ were detected and marked as c(SeO_4_^2−^)_2_ and c(SeO_3_^2−^)_2_. HPLC-ICPMS was performed on a PerkinElmer NexION2000 ICPMS coupled with Flexar20 HPLC system (PerkinElmer, MA, USA), using a Hamilton PRP-X100 column (Hamilton, MA, USA) as described by Cámara et al. (49). The content of SeO_4_^2−^, SeO_3_^2−^, and Se^2−^ in algal cells was calculated by equations 1-3, respectively.


(1) $$c\left({SeO}_{4}^{2-}\right)=c{\left({SeO}_{4}^{2-}\right)}_{1}$$

(2) $$c\left({SeO}_{3}^{2-}\right)=c{\left({SeO}_{3}^{2-}\right)}_{1}$$

(3) $$c\left({Se}^{2-}\right)=c{\left({SeO}_{4}^{2-}\right)}_{2}+c{\left({SeO}_{3}^{2-}\right)}_{2}-c{\left({SeO}_{4}^{2-}\right)}_{1}-c{\left({SeO}_{3}^{2-}\right)}_{1}$$

### Detection of thiamine and thiamine phosphates in algal cells

The analyses of thiamine, thiamine monophosphate, thiamine diphosphate, and thiamine triphosphate were performed according to Moulin et al. (50). After extraction and derivatization, samples were separated on an Agilent Eclipse XDBC18 column (4.6 × 250 mm, 5 µm pore size; Agilent, CA, USA) using an Agilent 1260 Infinity series HPLC system. For fluorescence detection, the excitation wavelength was 366 nm and the emission wavelength was 440 nm. Chemical standards were thiamine (Taitan, Shanghai, China), TMP (Taitan, Shanghai, China), TDP (Aladdin, Shanghai, China), and TTP (TRC, Toronto, Canada).

## Data Availability

The raw sequence data of RNA-Seq are deposited in the National Center for Biotechnology Information (NCBI) under the accession number SAMN21393911, which is associated with the BioProject number PRJNA762467.

## SUPPLEMENTAL MATERIAL

Table S1 mbio.02780-23-s0001.xlsx Summary of high-throughput sequencing data.

Table S2 mbio.02780-23-s0002.xlsx Summary of *de novo*-assembled unigenes.

Table S3 mbio.02780-23-s0003.xlsx DEGs of *S. hainanensis* X1 under heat stress.

Table S4 mbio.02780-23-s0004.xlsx DEGs of *Effrenium* sp. S1 under heat stress.

Table S5 mbio.02780-23-s0005.xlsx Summary of RT-qPCR experiments of *S. hainanensis* X1

Table S6 mbio.02780-23-s0006.xlsx Summary of RT-qPCR experiments of *Effrenium* sp. S1.

Table S7 mbio.02780-23-s0007.xlsx Temperature setup of high-throughput sequencing.

ASM does not own the copyrights to Supplemental Material that may be linked to, or accessed through, an article. The authors have granted ASM a non-exclusive, world-wide license to publish the Supplemental Material files. Please contact the corresponding author directly for reuse.
